# Mutational landscape and in silico structure models of SARS-CoV-2 spike receptor binding domain reveal key molecular determinants for virus-host interaction

**DOI:** 10.1186/s12860-021-00403-4

**Published:** 2022-01-07

**Authors:** Shijulal Nelson-Sathi, P. K. Umasankar, E. Sreekumar, R. Radhakrishnan Nair, Iype Joseph, Sai Ravi Chandra Nori, Jamiema Sara Philip, Roshny Prasad, K. V. Navyasree, Shikha Ramesh, Heera Pillai, Sanu Ghosh, T. R. Santosh Kumar, M. Radhakrishna Pillai

**Affiliations:** 1 Corona Research & Intervention Group, Rajiv Gandhi Centre for Biotechnology, Thiruvananthapuram, 695014 India; 2 SAGENOME Private Limited, BioNest, Kochi, 683503 India

**Keywords:** SARS-CoV-2, Spike protein, Receptor binding domain, Mutations, ACE2, Binding interface

## Abstract

**Background:**

SARS-CoV-2, the causative agent of COVID-19 pandemic is a RNA virus prone to mutations. Formation of a stable binding interface between the Receptor Binding Domain (RBD) of SARS-CoV-2 Spike (S) protein and Angiotensin-Converting Enzyme 2 (ACE2) of host is pivotal for viral entry. RBD has been shown to mutate frequently during pandemic. Although, a few mutations in RBD exhibit enhanced transmission rates leading to rise of new variants of concern, most RBD mutations show sustained ACE2 binding and virus infectivity. Yet, how all these mutations make the binding interface constantly favourable for virus remain enigmatic. This study aims to delineate molecular rearrangements in the binding interface of SARS-CoV-2 RBD mutants.

**Results:**

Here, we have generated a mutational and structural landscape of SARS-CoV-2 RBD in first six months of the pandemic. We analyzed 31,403 SARS-CoV-2 genomes randomly across the globe, and identified 444 non-synonymous mutations in RBD that cause 49 distinct amino acid substitutions in contact and non-contact amino acid residues. Molecular phylogenetic analysis suggested independent emergence of RBD mutants. Structural mapping of these mutations on the SARS-CoV-2 Wuhan reference strain RBD and structural comparison with RBDs from bat-CoV, SARS-CoV, and pangolin-CoV, all bound to human or mouse ACE2, revealed several changes in the interfacial interactions in all three binding clusters. Interestingly, interactions mediated via N487 residue in cluster-I and Y449, G496, T500, G502 residues in cluster-III remained largely unchanged in all RBD mutants. Further analysis showed that these interactions are evolutionarily conserved in sarbecoviruses which use ACE2 for entry. Importantly, despite extensive changes in the interface, RBD-ACE2 stability and binding affinities were maintained in all the analyzed mutants. Taken together, these findings reveal how SARS-CoV-2 uses its RBD residues to constantly remodel the binding interface.

**Conclusion:**

Our study broadly signifies understanding virus-host binding interfaces and their alterations during pandemic. Our findings propose a possible interface remodelling mechanism used by SARS-CoV-2 to escape deleterious mutations. Future investigations will focus on functional validation of in-silico findings and on investigating interface remodelling mechanisms across sarbecoviruses. Thus, in long run, this study may provide novel clues to therapeutically target RBD-ACE2 interface for pan-sarbecovirus infections.

**Supplementary Information:**

The online version contains supplementary material available at 10.1186/s12860-021-00403-4.

## Background

The Severe Acute Respiratory Syndrome Coronavirus-2 (SARS-CoV-2) has brought in a new normal to the world, by causing the COVID-19 disease [1]. It has already curbed many lives to date and the emerging new variants have also become a matter of great concern [2]. COVID-19 is a kind of pneumonia that affects the respiratory system, in severe cases cause hypoxemia and respiratory failure [3]. It has been reported that the disease spreads through the aerosols released from an infected individual while coughing, sneezing, and talking, etc. The spread of the disease occurs when these infected droplets are inhaled by a healthy individual. Moreover, the disease is also shown to spread through the fomites of the patient [4].

The SARS-CoV-2 belongs to the Sarbecovirus subgenus of Coronaviridaefamily. The other members of the family include the SARS-CoV, MERS-CoV, bat-CoV, pangolin-CoV and other endemic human coronaviruses [5]. The SARS-CoV-2, in specific, has four structural and 16 non-structural proteins that are important for viral replication and propagation. The structural proteins include the Spike protein (S-protein), Membrane protein (M-protein), Envelope protein (E-protein), and Nucleocapsid protein (N-protein), and the non-structural proteins include Nsp 1–16 [6].

The S-protein is responsible for viral entry; thus, has been the main target of diagnostics and therapeutics for COVID-19 [7, 8]. This homo-trimeric transmembrane protein is bipartite consisting of S1 and S2 subunits [9]. The virus uses S1 and S2 subunits to bind to host and to fuse to the host cell membrane [10]. The fusion occurs after cleavage via one of the host proteases- TMPRSS2 at cell surface, cathepsin-L in endolysosomes or furin like enzymes during trafficking in the producer cell [11]. These sequential steps ultimately facilitate SARS-CoV-2 entry into the respiratory system [12].

To initiate viral entry, a region in S1, spanning from Arg319 to Phe541 called Receptor Binding Domain (RBD) must interact with the N-terminal peptidase domain of Angiotensin Converting Enzyme-2 (hACE-2).

3-D structures of S-protein trimer or RBD of the SARS-CoV-2 Wuhan reference strain bound to human ACE2 has been extensively elucidated via X-ray crystallography [13, 14] cryo-EM [9, 15–18] and MD simulations [19–21]. These structures provided valuable clues regarding molecular architecture of the binding interface. A total of 21 contact residues were identified on RBD which interacts with 20 residues of the ACE2 peptidase domain. Most of these ACE2 engaging residues were found to be confined to a variable loop region within RBD called Receptor binding motif (RBM) [9, 13, 15].

SARS-CoV-2 genome has been predicted to mutate with ~ 1.12 × 10^−3^nucleotide substitutions per site per year [22, 23]. Mutational landscape of SARS-CoV-2 after an year of pandemic revealed considerable changes in the original Wuhan strain with 27 proteins mutating at different rates [24]. Among these, S-protein has been identified to be one of the highly mutated proteins with 4% mutations observed in the first quarter of the pandemic as reported in Koyama et al., 2020 [25] and in our own study. Significantly, some of these S-protein mutations dominated and contributed to the emergence of new variants of concern globally. For instance, the mutations ΔH69–V70, ΔY144, N501Y, A570D, D614G, P681H, T716I, S982A, and D1118H were associated with the alpha variant B.1.1.7 in United Kingdom [26]; S13I, W152C, L452R, and D614G with the epsilon variant B.1.429 in United States [27]; ΔL242–L244, L18F, D80A, D215G, R246I, K417N, E484K, N501Y, D614G, and A701V with the beta variant B.1.351 in South Africa [28]; L18F, T20N, P26S, D138Y, R190S, K417T, E484K, N501Y, D614G, H655Y, T1027I and V1176F with the gamma variant P.1 in Brazil [29], and T19R, V70F, T95I, G142D, E156G, F157G, R158G, A222V, W258L, K417N, L452R, T478K, D614G, P681R, D950N, E484Q and L452R with the delta variant B.1.617 in India [30, 31].

A few S-protein mutations have also been experimentally demonstrated to significantly improve virus transmissibility and immune evasion [2]. Experiments with the lung cell line Calu-3 showed that the D614G, a mutation outside the RBD region can induce conformational changes in S-protein which render enhanced stability for ACE2 binding leading to increased viral fitness and infectivity [32–36]. Similarly, mutations within the RBD- N439K, Y453F, S477N, E484K and N501Y, have also been shown to increase ACE2 binding affinity and improved viral transmissibility in humans and mink [37–39]. In addition, SARS-CoV-2 bearing N501Y, L452R and E484K mutations which overlap with major epitope regions on RBD have been shown to escape from highly neutralizing COVID-19 convalescent plasma [2, 40, 41].

Several independent studies reporting the mutational landscape of SARS-CoV-2 S- protein suggested that majority of mutations accumulated on RBD could be neutral in nature [42–47] likely favouring sustained viral spread during the pandemic [48, 49]. But, the structural basis for this neutral effect is currently unclear. We ask the following questions. What type of mutations accumulate on SARS-CoV-2 RBD. What are the molecular changes induced by these mutations on the binding interface. Can we gain valuable insights into the structural mechanism of RBD-ACE2 interface formation in SARS-CoV-2 and in other sarbecoviruses. Hence, in this study, we set out to investigate the possible structural mechanism behind RBD mutation effect. We have used high-fidelity bioinformatics pipeline, in silico- structure modelling/mutagenesis and molecular dynamic (MD) simulations, to analyze RBD mutations and corresponding structural rearrangements in the binding interface in the first six months (January–June 2020) of the pandemic.

## Results and discussion

### Non-synonymous RBD mutational profile

**Fig. 1:**
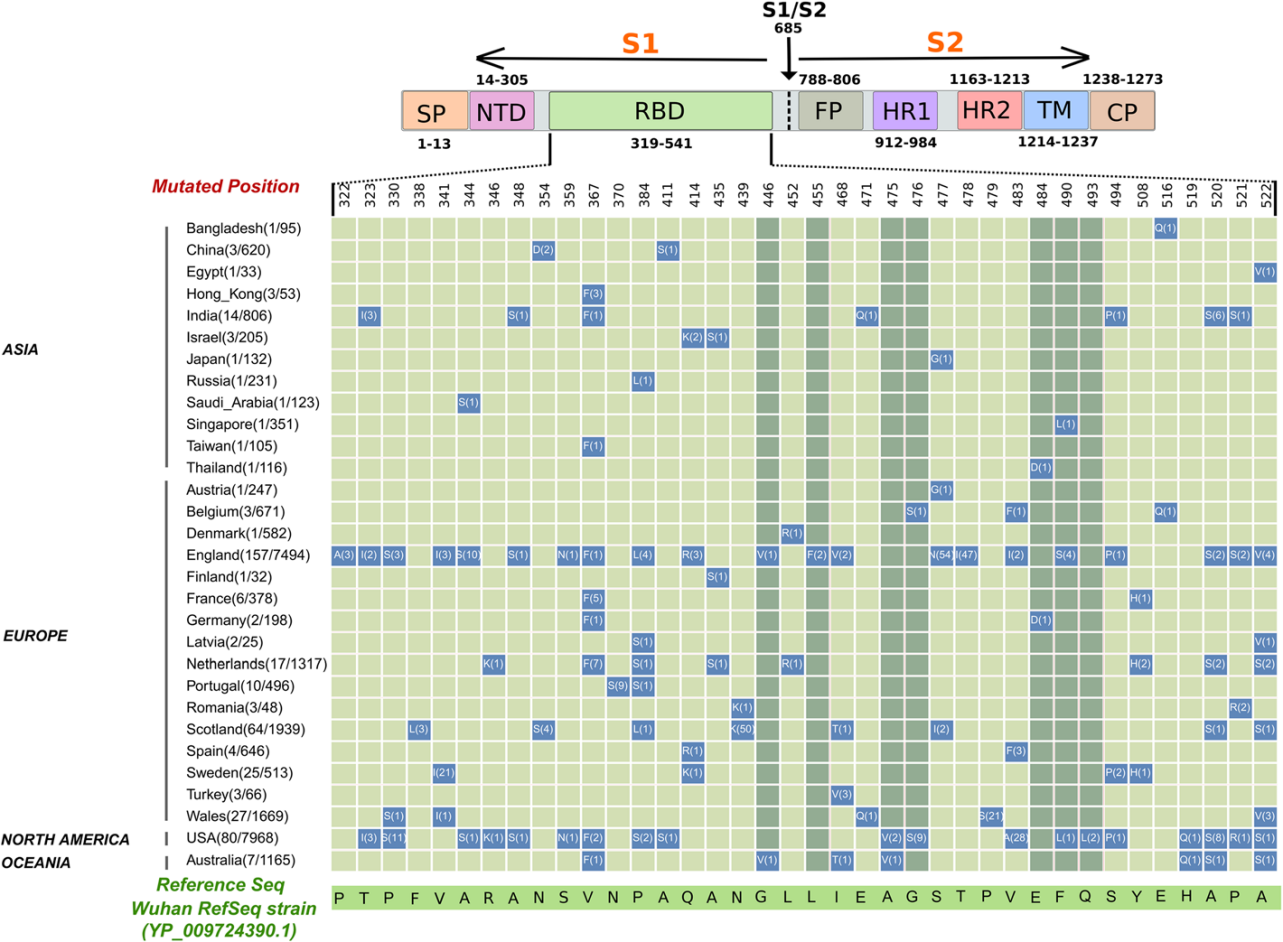
Matrix representing amino acid substitutions present in RBD domain of SARS-CoV-2 S protein of 31,403 genomes. Name of countries and the number of mutants vs. genomes sampled are given on the Y-axis and the relevant amino acid residues (single letter code) in the reference strain are given on the X-axis. Mutated amino acid residues and their frequency of occurrence are provided in matrix cells. Light green colored matrix cells represent non- interface mutations and dark green color matrix cells represent interface-mutations in the RBD domain of spike protein. Mutations, which are present, at least in two independent genomes at the same position are represented in the matrix along with their positions

To capture mutations that affect binding interface, we searched for non-synonymous mutations in RBD sequences from SARS-CoV-2 genomes. Using unbiased and stringent filtering criteria, we analyzed 31,403 genomes deposited in GISAID till 29th June 2020. Altogether, 444 non-synonymous mutations in RBD were identified that belong to viral genomes from 30 countries. Overall, RBD mutations accounted for ~ 9% of the total non-synonymous mutations in S-protein. These mutations were found to substitute 49 amino acid residues in which 23 residues lie within RBM (Fig. 1). These include contact residues that directly engage ACE2 (G446, L455, A475, G476, E484, F490 and Q493) and non-contact residues that are present in the near binding vicinity. Hot spot mutations were also identified that caused recurrent substitutions of amino acid residues in the same position (N354, P384, Q414, I468, S477, V483, F490, A520, P521 and A522). Each RBD mutation was found to be unique to the genome; a combination of mutations was never observed in our analysis.

### Evolutionary pattern of RBD variants

**Fig. 2:**
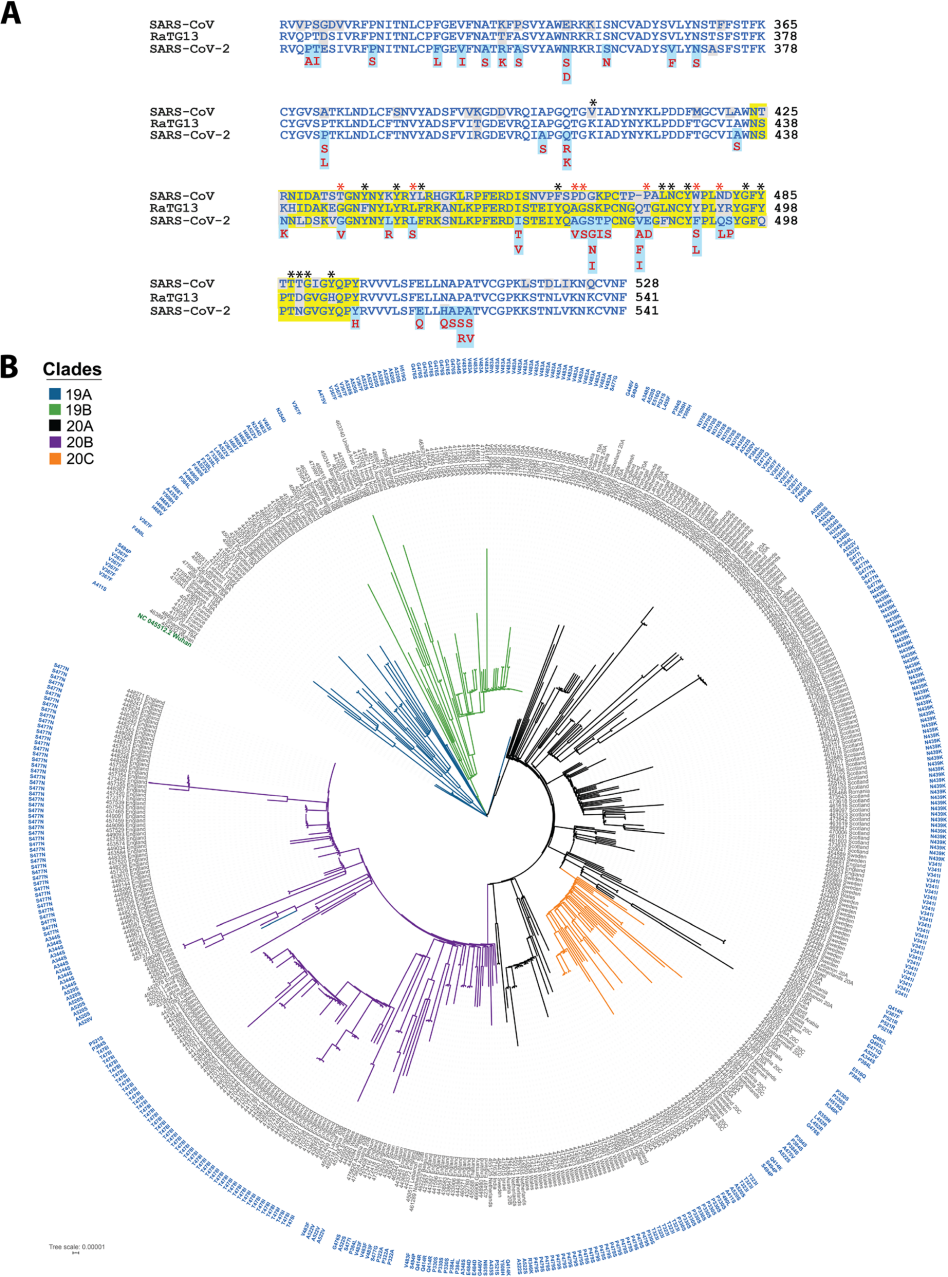
(**A**) Conservation of Receptor Binding Domain (RBD) of SARS-CoV-2 with its close relatives, SARS-CoV and Bat RaTG13. The blue colored region shows RBD and the yellow highlighted region within RBD is the Receptor Binding Motif (RBM). The mutated residues are highlighted in light blue and substitutions are marked below. Non-conserved residues are highlighted in grey color. Interacting residues are marked with black asterisk and the mutated interactions are in red asterisk symbol. (**B**) The carton model representation of SARS-CoV-2 RBM highlighting mutated interacting residues and most frequent mutations (red color) in RBM. (**C**) Maximum Likelihood Phylogenetic tree of 494 SARS-CoV-2 isolates containing RBD mutations. The outer circle represents the RBD mutations

To see the evolutionary trend in RBD mutations, we compared RBDs from SARS-CoV-2, the related SARS-CoV and the bat coronavirus RaTG13, a sister lineage of SARS-CoV-2. SARS-CoV-2 RBD is 73.4% identical to SARS-CoV and 90.1% identical to RaTG13 [50, 51] (Fig. 2A).

We identified several RBD mutations on residues that are unique to SARS-CoV-2 (N439K, V483A/F/I, E484D, F490S/L, Q493L and S494P) or are conserved in all three viruses. In addition, we observed mutations in SARS-CoV-2 that interchange residues to that in SARS-CoV or RaTG13 (R346K, N354D, N439K, L452R, E471V and S477G). Interestingly, most of these reversion mutations were located in the RBM region and thus may have implications in viral tropism [30, 37]. We performed phylogenomic analysis to understand the evolutionary pattern of RBD variants during pandemic. In the phylogenetic tree, we observed an unbiased distribution of RBD variants among distinct SARS-CoV-2 clades (19A, 19B, 20A, 20B, 20C). This likely indicates independent emergence of these mutants during pandemic (Fig. 2B).

Interestingly, L452R, R346K and F490S mutations observed in our study sustained in delta, mu and lambda variants respectively which evolved later during the pandemic. However, E484D mutation which likely did not affect viral infectivity in the beginning of pandemic, evolved with an acidic to basic residue change (E484K) in beta, gamma and mu variants and then with a neutral residue (E484Q) in the delta variants. Likewise, T478I mutation evolved to T478K in the delta variants of concern. Overall, these findings suggest the role of RBD residues in shaping a unique pattern for SARS-CoV-2 evolution.

### Structural implications of RBD mutations

**Fig. 3:**
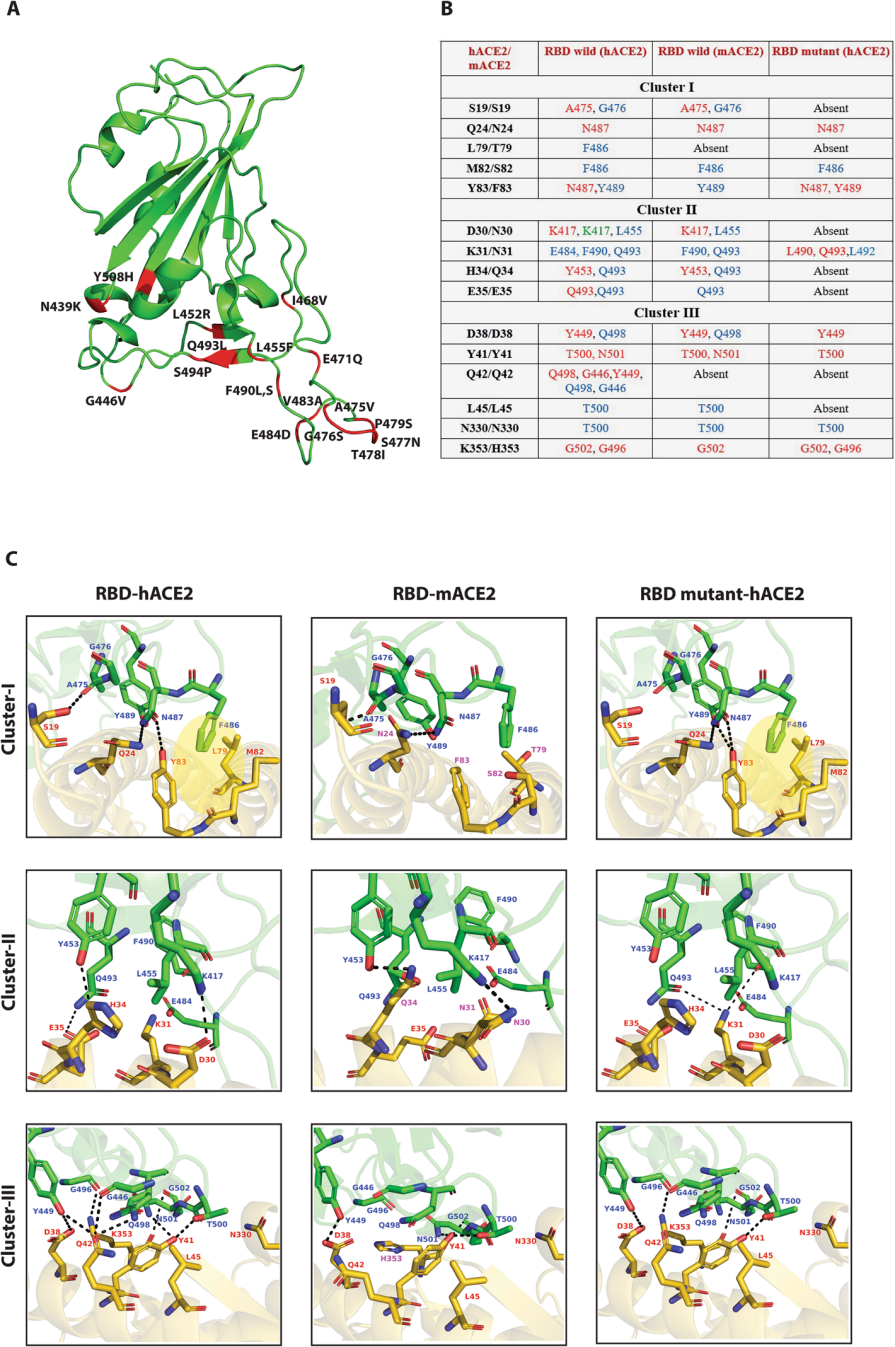
Molecular rearrangements in RBD-ACE2 interface. (**A**) The cartoon model representation of SARS-CoV-2 RBM highlighting mutated interacting residues and most frequent mutations (red color) in RBM. (**B**) List of cluster specific molecular interactions of hACE2, mACE2, and mutated RBD-ACE2 complexes. Hydrogen bonds are marked in red, van der Waal’s interactions in blue and salt bridges in green. (**C**) Structural visualization of key interactions listed in (B). RBD is represented in green and ACE2 in gold. The hydrogen bond interactions between ACE2 and RBD are shown as dotted lines

Structurally, RBM scaffold resembles a concave arch that makes three contact points with ACE2 α- helix; Cluster-I, II and III. Cluster-I and Cluster-III are on two ends and Cluster-II is towards the middle of the interface (Fig. 3A and B). It has been shown that certain adaptive mutations in the RBD binding residues of mouse ACE2 destabilize the interface rendering the organism resistant to infection from SARS-like coronaviruses [52]. Hence, to gain insights into relevant interactions that can create a stable interface, we included RBD-mouse ACE2 complex in our analysis. In this structural background, we analyzed the impact of RBM mutations on each of the binding clusters in SARS-CoV-2 by using two in silico approaches: structural modelling and mutagenesis (Fig. 3B, C and Additional File 1: Table S1).

Each mutation was modelled based on the reported crystal structures of SARS-CoV-2 RBD-ACE2 bound complex [14]. The key interactions that stabilize cluster-I in SARS-CoV-2 are formed between RBD:A475, G476-ACE2:S19; RBD:N487-ACE2:Q24, Y83 and RBD:F486-ACE2:L79, M82 [14]. We observed that RBD:A475, G476-ACE2:S19 and RBD:F486-ACE2:L79 interactions completely disappeared in several mutants (Fig. 3B, C and Additional File 1: Table S1). A475, G476 and F486 are considered critical hotspot residues for ACE2 binding [42]. Further mutations in A475 and G476 have been shown to weaken RBD-ACE2 binding [53]. Similarly, an adaptive mutation in the F486 interacting residue on ACE2 (L79T) abolished stable interface formation in mouse [52] **(**Fig. 3B, C**).** In addition, a natural F > L substitution in SARS-CoV prevents formation of a complete hydrophobic binding pocket in the interface leading to reduced ACE2 binding affinity and infectivity of the virus [54]. Thus, it appears that the disrupted cluster-I interactions in mutants may be critical for virus transmissibility [43, 44]. Intriguingly, a new hydrogen bond between RBD Y489-ACE2 Y83 was observed in cluster-I mutants. Also, RBD N487-ACE2 Q24, Y83 and RBD F486-ACE2 M82 interactions remained largely unaffected in all the mutants (Fig. 3B, C and Additional File 1: Table S1). Together, it suggests the possibility of compensatory mechanisms to maintain hydrophobicity in RBD cluster-I.

Cluster-II is stabilized by polar /charged residue interactions via hydrogen bonds, van der Waals forces and salt bridges. The major bonds form between RBD:K417, L455- ACE2:D30; RBD:E484-ACE2:K31; RBD:Y453- ACE2:H34 and RBD:Q493- ACE2:E35 [14]. Mutational studies have identified K417, L455 and E484 as enhancers of ACE2 binding [53, 55]. The presence of unique K417-D30 salt bridge in SARS-CoV-2 has been shown to significantly enhance receptor binding and infectivity [56]. Further, K417 salt bridge and E484 van der Waal’s interactions were abolished in mouse due to adaptive mutations on ACE2 (D30N, K31N) [52] (Fig. 3B, C). Surprisingly, these key interactions were missing in majority of mutants in our analysis (Fig. 3B, C and Additional File 1: Table S1). Nevertheless, interactions with ACE2 K31, a hotspot of binding for SARS-CoV-2 and SARS-CoV [10, 57, 58] were maintained in all the mutants. Here, the van der Waal’s forces in the reference Wuhan strain were found to be replaced with a new set of hydrogen bonds formed via Q493/L490 residues in these mutants likely strengthening the hotspot interactions (Fig. 3B, C and Additional File 1: Table S1).

Cluster-III interactions involving RBD:Y449, Q498- ACE2:D38; RBD:T500, N501-ACE2: Y41; RBD:Q498, G446- ACE2:Q42; RBD:T500- ACE2:L45, N330 and RBD:G502, G496- ACE2:K353 are known to model the binding interface in SARS-CoV-2. Several studies reported Q498 and N501 RBM residues as high affinity binders [14]. The absence of their hydrogen bond interactions in mouse interface further confirms the importance of these residues [52] (Fig. 3B, C). Moreover, single N501Y mutation showed 10-fold increase in ACE2 binding in the SARS-CoV-2 variants of concern [39]. Surprisingly, the cluster of interactions mediated by Q498 and N501 were absent in some mutants (Fig. 3B, C and Additional File 1: Table S1). A few mutants also exhibited partial disruption of T500 interactions with ACE2. However, interactions involving the ACE2 critical residues D38, Y41, N330 and the hotspot residue K353 were all retained in all the mutants indicating significance of these amino acids in shaping the interface (Fig. 3B, C and Additional File 1: Table S1). Thus our findings suggest that the binding interface of SARS-CoV-2 remodels constantly regardless of the position and number of RBD mutations.

### Stability of RBD-ACE2 complexes

**Fig. 4:**
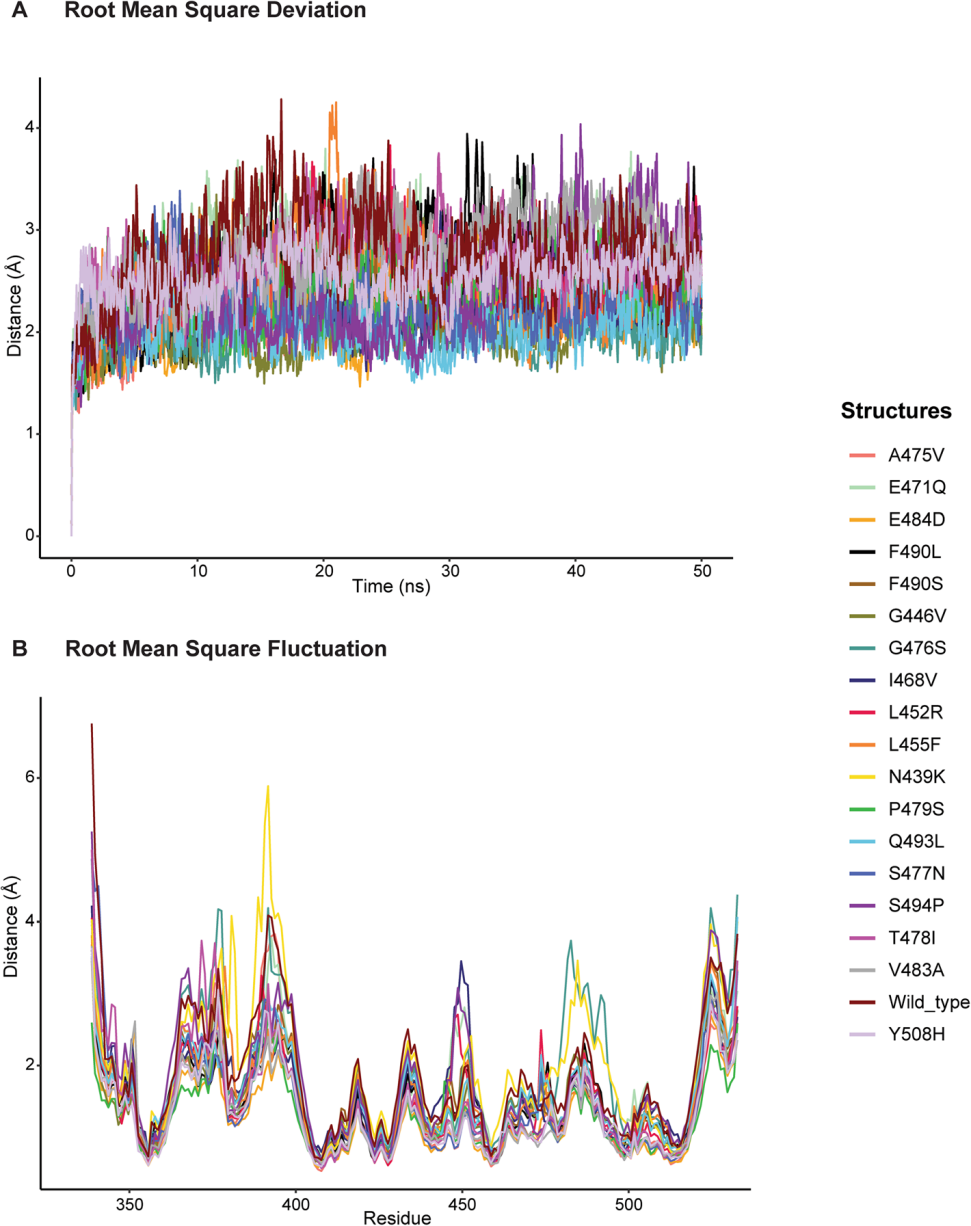
Structural stability of SARS CoV-2 wild type and mutant RBD –ACE2 complexes. (**A**) Root mean square deviation (RMSD) of wild type and mutant RBD with ACE2 complexes. (**B**) Root mean square fluctuation (RMSF) of RBD wild type and mutant structures. Each mutant and wild type are separately colour coded

MD simulations of wild type and mutated RBD- ACE2 complexes were carried out for 50 ns to analyse the stability. The root mean square deviation (RMSD) for each complex was calculated. RMSD was found to be below 3 Å in wild type and mutants suggesting good stability of the complexes during simulations (Fig. 4A). The fluctuations in each residue of RBD over time were also analysed by plotting the Root Mean Square Fluctuation (RMSF) graph (Fig. 4B). The β-sheets were less fluctuating throughout the simulation. The higher peaks were mainly observed in the loop regions in both wild type and mutant RBDs, indicating more fluctuations in loop regions than structured regions. However, the overall values were below 3 Å further suggesting that the RBD-ACE2 complexes remained stable and bound together throughout the simulation.

### Binding affinity of RBD mutants

**Fig. 5:**
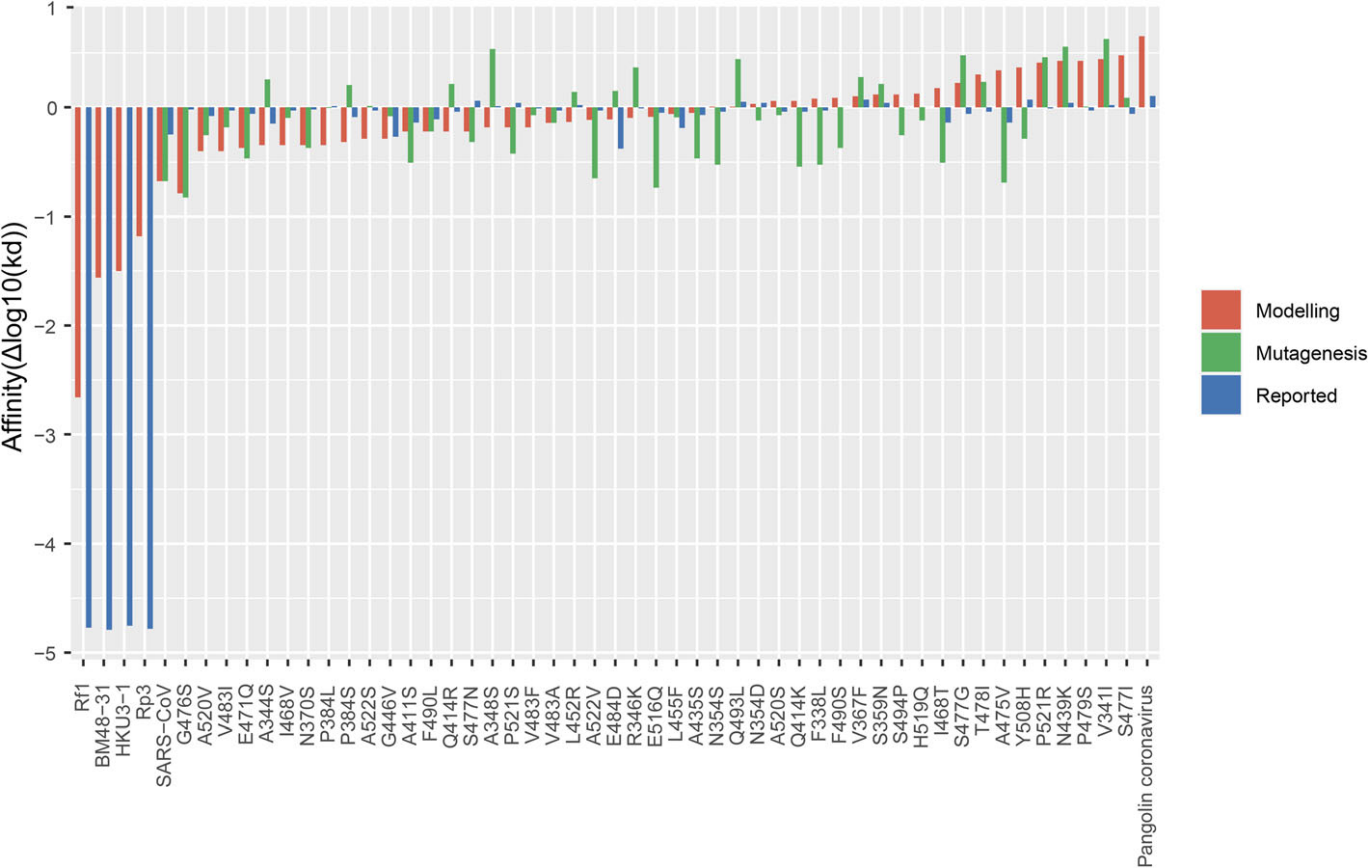
Bar graph representing variations in binding affinity differences among all RBD mutants and other coronaviruses. Orange, green and blue bars indicate Δlog10 (K_d_) values obtained from modelling, mutagenesis and functional studies reported

Binding affinities were derived from both modelled and mutated structures. For detailed comparison, we also calculated binding affinities from modelled RBDs of ACE2 dependent (SARS-CoV, pangolin-CoV) and ACE2 independent (BM48–31, Rf1, Rp3, HKU3–1) sarbecoviruses [59]. The differences in binding affinities with respect to wild type SARS-CoV-2 Δlog10 (K_d_) were calculated by the following equation and plotted as shown in Fig. 5:

1 $$ \Delta \log 10\ \left(\mathrm{KD}\right)=\log 10\ \left(\mathrm{KD}\right)\ \mathrm{wild}-\log 10\ \left(\mathrm{KD}\right)\ \mathrm{mutants} $$

SARS-CoV and pangolin CoV were on the two ends of the spectrum showing ~ 7 fold decrease and increase in the binding affinity respectively compared to wild type SARS-CoV-2. Δlog10 (K_d_) values of all observed RBD fall within this range with the lowest affinity mutant close to that of SARS-CoV and the highest affinity mutant close to pangolin CoV (Fig. 5). As expected, all the ACE2 independent sarbecoviruses showed 20-30fold lower Δlog10 (K_d_) values validating our analysis. The binding affinity differences (Δlog10 (KD) obtained from our analyses were comparable up to 60% with the experimental values reported in other studies [43, 44]. The 40% mismatch may attribute to differences in affinity, avidity and conformation of trimeric spike (used for experiments) versus monomeric RBD (used for *in-silico* analysis). We did not observe a significant correlation between binding energies and mutations in contact versus non-contact residues. This suggests that mutations on any RBM residues could impact spatial arrangements of backbone leading to altered binding affinities. Overall, our observation were consistent with recent studies showing that the whole RBM, and not the ACE2 binding residues alone, was necessary to complement viral entry of ACE2 independent sarbecoviruses [59, 60].

### Possible structural mechanism of RBD-ACE2 interface formation in sarbecoviruses

**Fig. 6:**
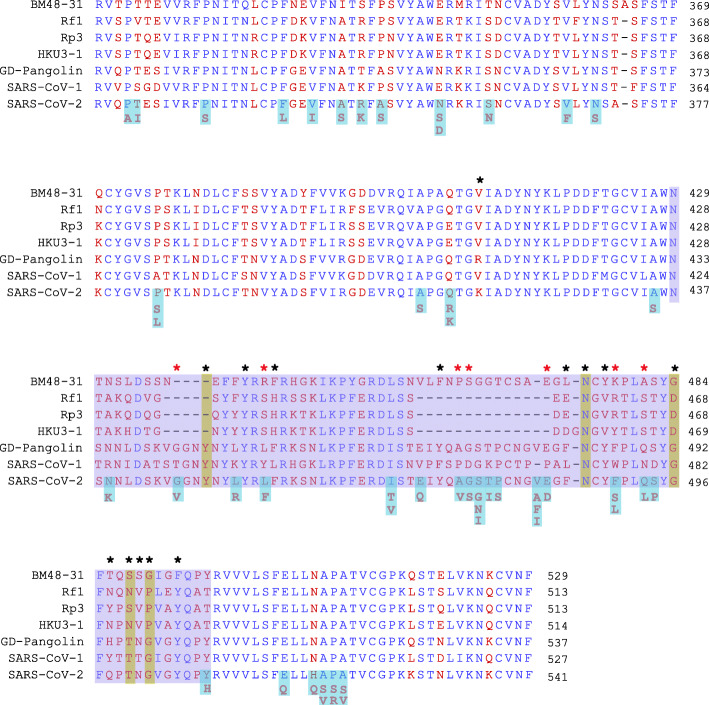
Multiple sequence alignment of RBD across sarbecoviruses. The blue highlighted box denotes RBM. Black asterisks indicate RBD residues that directly bind to ACE2. Red asterisks denote mutations on the binding residues analyzed in this study. The mutated residues are highlighted in light blue and substitutions are marked below. Binding residues in cluster-I, II and III are marked in red, green and blue bars on the top. 1A,B 2A,B and 3A,B indicates the RBD epitopes present in SARS-CoV-2

Based on our structural analysis of RBD mutants, we surmised that the RBD contact residues which remained unaffected in all the mutants- N487, F486 and Y489 residues in cluster-I, E484, F490 and Q493 and Y449, G496, T500, G502 in cluster-III could possibly play a crucial role in the formation of a stable binding interface. Given the spatial arrangement, these residues appear critical in directly anchoring the RBM loop to ACE2 from both ends. This may help initiate interface formation that structurally favours viral entry [61]. The significant changes in cluster-II interactions indicate they are dispensable for anchoring but might be important for remodelling the interface. Intriguingly, the corresponding residues on ACE2 (Q24, M82, Y83, D38, Y41, N330, K353) that interact with these RBD residues have been shown to be crucial for interspecies transmission of sarbecoviruses [61–63]. To understand structural evolution of RBD-ACE2 interface, we looked at the conservation of these residues across ACE2-dependent and ACE2-independent sarbecoviruses (Fig. 6). N487 was highly conserved in all sarbecoviruses whereas Y449, and T500 were present only in ACE2-dependent sarbecoviruses. Interestingly, Y489, G496, G502 and F486 which changed to L in SARS-CoV were found to be conserved in BM48–31. BM48–31 is a sarbecovirus distinct from other ACE2-independent viruses and likely shows evolution toward ACE2 dependency [64, 65]. A large deletion near RBM cluster-I responsible for disruption of ACE2 interaction is not present in BM48–31 [64]. In addition, E484 residue which is important for stabilizing cluster-II in SARS-CoV-2 and its replacement L492 observed in mutants are both conserved in BM48–31 (Fig. 6). Together, it supports possible evolution of a favourable ACE2 binding interface in this virus [65]. Many residues important in interface formation and remodelling are also part of distinct epitopes present on RBD. Significantly, mutations in A475, F486, E484 etc., have been shown to be immune evasive [41, 66]. Hence, interface remodelling mediated by these residues may help virus to simultaneously sustain ACE2 binding and escape neutralizing antibodies [55].

## Conclusions

Currently, all SARS-CoV-2 immunogens and testing reagents are based on the Wuhan reference sequence. Thus, growing number of mutations in the reference strain is wreaking a global havoc regarding efficacy of vaccines and therapeutics. Our elucidation of mutational landscape and the corresponding structural landscape is a novel approach to completely understand the virus and its interaction with the host. The predicted mechanism of interface remodelling in our study may be useful to design novel strategies to combat coronavirus infections in general. Overall, our study proposes the significance of understanding structural evolution of protein interfaces during pandemics.

## Materials and methods

### Mutational analysis

A total of 55,485 spike proteins of SARS-CoV-2 were directly downloaded on 29th June 2020 from the GISAID database. We removed the partial sequences, sequences greater than 1% unidentified ‘X’ amino acids and sequences from low quality genomes. Further, 31,403 spike protein sequences along with Wuhan reference spike protein (YP_009724390.1) were aligned using Mafft (maxiterate 1000 and global pair-ginsi) [67]. The alignments were visualized in Jalview [68] and the amino acid substitutions in each position were extracted using custom python script. We ignored the substitutions that were present in only one genome and unidentified amino acid X. The mutations that are present in at least two independent genomes in a particular position were further considered. These two criteria were used to avoid mutations due to sequencing errors. The mutated amino acids were further tabulated and plotted as a matrix using R script.

### Phylogeny reconstruction

For the Maximum-likelihood phylogeny reconstruction, we have used the SARS-CoV-2 genomes containing RBD mutations, and 10 genomes were sampled as representatives for each known subtype with Wuhan RefSeq strain as root. Sequences were aligned using Mafft (maxiterate 1000 and global pair-ginsi), and phylogeny was reconstructed using IQ-Tree [69]. The best evolutionary model (GTR + F + I + G4) was picked using the Model Finder program [70].

### Structural analysis

The structural analysis of the mutated spike glycoprotein of SARS-CoV-2 RBD domain was done to assess the impact of interface amino acid residue mutations on binding affinity towards the human ACE2 (hACE2) receptor. The crystal structure of the SARS-CoV-2 RBD-hACE2 receptor complex was downloaded from Protein Data Bank (PDB ID: 6LZG) and the mutagenesis analysis was performed using Pymol [71]. As an alternative approach, we also modelled the mutants of SARS-CoV-2 RBD-ACE complex and other coronaviruses spike RBD bound with hACE2 receptors using Swiss model [72]. In addition, homology modelling of Mouse ACE2 (mACE2) structure was performed in Swiss-Model using SARS-CoV-2 RBD-hACE2 as template. The YASARA server [73] was used for the energy minimization of analysed structures. The Z-dock webserver [74] was used for docking the mACE-2 and the spike protein RBD of SARS-CoV-2. The binding affinity of the wild, mutated and docked structures was calculated using PRODIGY web server [75]. The hydrogen bond and salt bridge interactions were calculated using Protein Interaction Calculator [76] and the van der Waals interactions were calculated using Ligplot [77]. All the visualizations were done using Pymol [71].

### MD simulations

The stability of the wild type and mutated structures were analysed by Molecular Dynamic (MD) Simulations using Desmond (Desmond, Schrödinger, LLC, NY, USA) [78]. The wild type and mutated SARS CoV-2 RBD – ACE2 complex were prepared by Schrodinger Maestro Protein Preparation wizard. The water molecules were removed and optimized the structures by adding Hydrogen atoms. The system was solvated using TIP3P water model and neutralized by adding Na/Cl ions and minimized using OPLS3e force field. The Nose-Hoover chain thermostat method and Martyna-Toubias-Klein barostat method were used to maintain the temperature and pressure of the system respectively. A 50-ns simulation for each mutant and wild type RBD-ACE2 complex were done in an NPT Ensemble of 300 K at 1.01325 bar.

## Supplementary Information

**Additional file 1: Table S1.** List of clusterwise interfacial interactions between wild type or mutant SARS CoV-2 RBD protein and human or mouse ACE2.

## Data Availability

The SARS-CoV-2 datasets analysed during the current study are available in the GISAID-EpiCoV databases. (https://www.gisaid.org/).

## Abbreviations

S: Spike
RBD: Receptor Binding Domain
ACE2: Angiotensin-Converting Enzyme 2
RBM: Receptor binding motif
hACE2: human ACE2
mACE2: Mouse ACE2
